# Evolutionary Comparison of Two Combinatorial Regulators of SBP-Box Genes, *MiR156* and *MiR529*, in Plants

**DOI:** 10.1371/journal.pone.0124621

**Published:** 2015-04-24

**Authors:** Shu-Dong Zhang, Li-Zhen Ling, Quan-Fang Zhang, Jian-Di Xu, Le Cheng

**Affiliations:** 1 Germplasm Bank of Wild Species, Kunming, 650201, China; 2 BGI-Yunnan, BGI-Shenzhen, Kunming, 650106, China; 3 Bio-Tech Research Center, Shandong Academy of Agricultural Sciences, Jinan 250100, China; 4 Shandong rice research institute, Shandong Academy of Agricultural Sciences, Jinan, 250100, China; Universidade Federal do Rio Grande do Sul, BRAZIL

## Abstract

A complete picture of the evolution of miRNA combinatorial regulation requires the synthesis of information on all miRNAs and their targets. *MiR156* and *miR529* are two combinatorial regulators of *squamosa* promoter binding protein-like (SBP-box) genes. Previous studies have clarified the evolutionary dynamics of their targets; however, there have been no reports on the evolutionary patterns of two miRNA regulators themselves to date. In this study, we investigated the evolutionary differences between these two miRNA families in extant land plants. Our work found that *miR529* precursor, especially of its mature miRNA sequence, has a higher evolutionary rate. Such accelerating evolution of *miR529* has significantly effects on its structural stability, and sequence conservation against existence of itself. By contrast, *miR156* evolves more rapidly in loop region of the stable secondary structure, which may contribute to its functional diversity. Moreover, *miR156* and *miR529* genes have distinct rates of loss after identical duplication events. *MiR529* genes have a higher average loss rate and asymmetric loss rate in duplicated gene pairs, indicating preferred *miR529* gene losses become another predominant mode of inactivation, that are implicated in the contraction of this family. On the contrary, duplicated *miR156* genes have a low loss rate, and could serve as another new source for functional diversity. Taken together, these results provide better insight into understanding the evolutionary divergence of *miR156* and *miR529* family in miRNA combinational regulation network.

## Introduction

MicroRNAs (miRNAs) are small, non-coding RNA molecules that regulate gene expression by binding to target mRNA transcripts, leading to either translational repression or mRNA degradation. A growing body of evidence indicates that, in plants, a single miRNA can target and regulate multiple transcripts and conversely, the same genes can be targeted by a number of different miRNAs [1–3]. With the identification of thousands of miRNAs through high-throughput sequencing, many transcription factors (TF), such as MYB, *APETALA2* (*AP2*), and MADS-box gene families, have been shown to be regulated by distinct miRNAs in plants [1,3]. However, we still have no idea about how the combinations of these different miRNAs work in concert to repress a target gene. In order to address this question, the basic principles that underlie the respective evolution of individual miRNA regulators and their target genes must be clarified firstly.

*MiR156* and *miR529* have been demonstrated to cooperatively target squamosa promoter binding protein-like (SBP-box) genes [4]. They share a 14–16-nt-long homologous stretch in their mature sequences and have overlapping binding sites in the same target [5,6]. Our previous study showed that their targeted SBP-box genes differ greatly in their evolutionary patterns and dynamics [6]. Whether there exist evolutionary differences between *miR156* and *miR529* is still a mystery in plants until now. However, we found that *miR156* and *miR529* are significantly different in three aspects. First, analysis of miRNA deep sequencing expression profile from rice has shown that *miR156* genes are ubiquitously expressed in the root, shoot and other tissues at the seedling stage, whereas *miR529* genes are only detected in the panicle throughout development [1]. A similar phenomenon was also found in maize and *Brachypodium distachyon*, suggesting that ancient *miR156* and *miR529* genes have formed conserved expression patterns over long evolutionary periods [7,8]. Second, extensive genomic analysis showed that *miR156* is ubiquitous throughout land plants and its regulatory circuit is extremely well conserved throughout plant evolution [9,10]. By contrast, *miR529* is only present in bryophytes, lycopods, and monocots, and displays limited taxonomic distributions [11,12]. These findings indicate that *miR529* is at least 400 million years old and may have been lost from all core eudicots and that the evolutionary forces driving the loss of this regulatory system are intriguing. Finally, *miR529* is decreased in monocots compared to moss, whereas *miR156* is increased in number [6]. This phenomenon means that there was dramatic expansion of the *miR156* family and sharp contraction of the *miR529* family during plant evolution. The aforementioned differences between *miR156* and *miR529*: expression pattern, taxonomic distribution, and the number of members in the miRNA family hinted that these two miRNA families may have undergone different evolutionary pathways.

The aim of this study was to explore the evolutionary differences between *miR156* and *miR529* families from moss to flowering plants. By comparing their evolutionary rate, thermodynamic stability, sequence conservation, and rate of gene loss after gene duplication, we will reveal the differences of evolutionary dynamics between *miR529* and *miR156*. And, the correlations between the different evolutionary dynamics of these two miRNA families and their observed differences in extant land plants will be also discussed.

## Materials and Methods

### Sequences of *miR156* and *miR529* in plants

Although over 300 *miR156* sequences are registered in miRBase database (release 21) [11], only 43 high-confidence entries were collected for this study. It is reported that *miR529* exists in several land plant organisms, but some *miR529* genes had been obtained by similarity search and had not been validated with sufficiently experimental evidence prior to their addition to miRBase. To eliminate potential inaccuracies, these *miR529* sequences were excluded from our analyses. Finally, a total of 11 *miR529* members were selected from four plant species: *Physcomitrella patens*, *Oryza sativa* subsp. *japonica*, *Brachypodium distachyon*, and *Zea mays*. All precursor and mature sequences of *miR156* and *miR529* were downloaded from miRBase (release 21) [11]. The information on *miR156* and *miR529* genes used in this study is summarized in S1 Table.

### Evaluation of substitution rate

The precursor sequences of *miR156* and *miR529* were separately aligned using ClustalX [13] and adjusted manually. The refined precursor alignments were used to calculate substitution rates using MEGA with the Kimura 2-parameter model [14,15]. Previous studies indicated that considerable heterogeneity exists in the relative rates of evolution of different regions of the precursor miRNA sequences [16,17]. Therefore, we parsed these precursor sequences into four regions as described by Shen [18]: mature miRNA, miRNA complement (miRNA*), loop end, and stem extension (a stem structure, beyond the Dicer cut site). The substitution rate of each constituent part was calculated with the method described above. The independent samples t-test is used for statistical significance.

### Computation of folding energy and sequence identity of *miR156* and *miR529* precursors

RNAfold [19] was used to compute the minimal free energy (MFE) of each *miR156* and *miR529* precursor structure using default settings. Moreover, we eliminated the influence of the length of the miRNA precursor sequence by normalizing MFE as previously described [20]. In this method, the normalized MFE (NMFE) was defined as the MFE divided by the sequence length of the miRNA precursor.

The sequence identity of precursor alignments of *miR156* and *miR529* was estimated via the online software Clustal Omega (http://www.ebi.ac.uk/Tools/msa/clustalo/) with the default setting. The same method was also applied to calculate sequence identify of mature alignments of *miR156* and *miR529*.

### Detecting *miR156* and *miR529* expansions, dating duplication events, and estimating rates of gene loss

Tandem duplication and segmental duplication significantly contribute to gene family expansion. Thus, we mainly focused on these two patterns of gene expansion in this study. Tandem duplications are characterized as multiple members of a gene family occurring within the same or neighboring intergenic regions and segmental duplications are defined as segments of DNA with near-identical sequence that map to two or more genomic locations. Of the four organisms analyzed in this study, we chose rice as the representation for analyzing *miR156* and *miR529* gene duplications because it has no less than two members in each miRNA family and a good genome annotation. Segmental duplications of *miR156* and *miR529* genes in rice genomes were retrieved from the Plant Genomic Duplication Database (PGDD) dataset [21]. In this dataset, many protein-coding gene pairs excluding genes encoding miRNAs were catalogued in each duplicated block. In theory, if the members of a miRNA family reside within a duplicated block, then neighboring protein-coding genes would also be present on the same duplicated block [22]. We therefore used 10 protein-coding genes both upstream and downstream of each *miR156* and *miR529* gene as guides to identify the duplicated block in which a *miR156* or *miR529* gene resides. The syntenic relationship between each miRNA gene pair in the duplicated block was visualized using MicroSyn [23].

The rates of synonymous substitution (Ks) of duplicated genes are expected to be similar over time [24]. Therefore, we used Ks values to estimate the time at which the segmental duplication events took place. The Ks value of each gene pair within a duplicated block was extracted from the PDGG dataset [21]. The mean Ks value was calculated and used to date the duplication events. In this analysis, any Ks values greater than 2 were discarded because of the risk of saturation [16]. The approximate time at which the duplication event took place was then calculated using the mean Ks and an estimated rate of silent-site substitutions of 6.5×10^–9^ substitutions/synonymous site/year [25].

The rate of gene loss was estimated according to Wang’s method [26]. The two copies in a duplicated block are referred to as copy 1 and copy 2 and the rate of gene loss in copy 2 was estimated as follows:

$$\frac{\text{S}1}{\text{S}1+\text{N}2}\times 100\%$$

Where, S1 is the number of single-copy genes in copy 1; N2 is the number of the extant genes in copy 2.

## Results

### A higher evolutionary rate of *miR529* in the mature sequence

**Fig 1:**
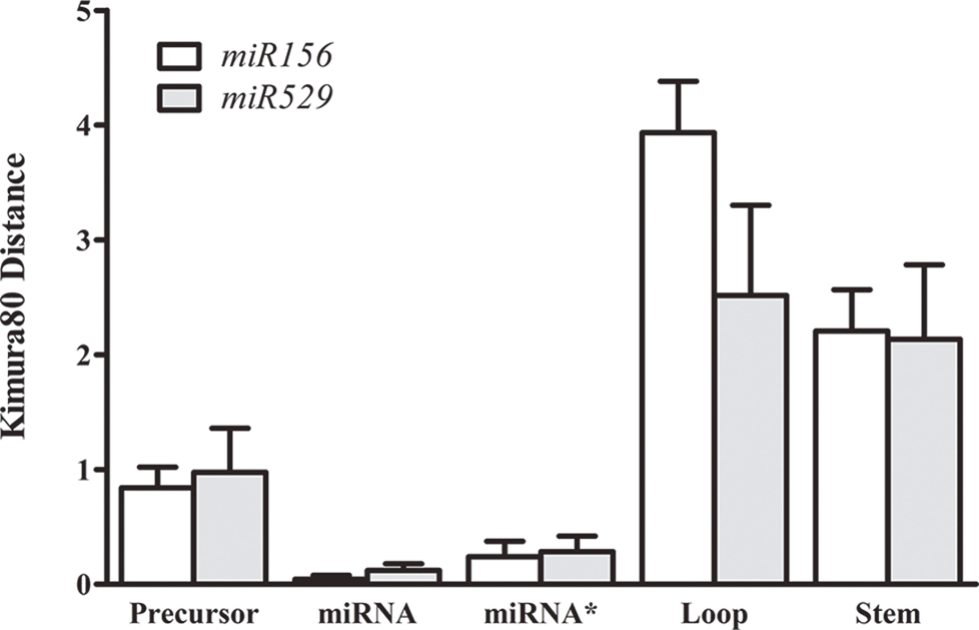
Comparison of evolutionary rates of *miR156* and *miR29* precursor sequences and their four structural elements. Error bars indicate the standard error of the mean.

It has been reported that ubiquitously expressed genes evolve more slowly than tissue-specific genes, which suggests that the extent to which genes are expressed is critical for their evolutionary rates in multicellular organisms [27]. Therefore, we conjectured that narrowly expressed *miR529* genes might have higher evolutionary rate than broadly expressed *miR156* genes. To test this hypothesis, we first measured the evolutionary rates of the precursor miRNA (pre-miRNA) between the two miRNA families using MEGA with the Kimura 2-parameter model [14,15]. The result shows that the average evolutionary rate of *miR529* precursor sequences was higher than that of *miR156* precursor sequences; however the differences were not statistically significant (p>0.05). Subsequently, we measured the independent evolutionary rates for the four parts of the precursor miRNA. In general, mature miRNA, as the functional part of the precursor miRNA, was well conserved between distant lineages and fewer mutations were found within its sequence, while the loop was the most variable region of the precursor sequence. As may be expected, the evolutionary rate was highest in the loop-containing region in both *miR156* and *miR529* families (Fig 1). Interestingly, the observed evolutionary rate in this region was significantly lower in the *miR529* family members than in the *miR156* family members (p<0.01). The evolutionary rates in the stem and miRNA* regions were comparable between both miRNA families. Unexpectedly, the evolutionary rate of *miR529* sequences was two times higher than that of *miR156* sequences in the mature miRNA region (p<0.01), which is contrary to our findings in the loop region. Collectively, these data suggest that *miR529* has higher evolutionary rate in precursor, especially in mature sequences, whereas an accelerating evolution of *miR156* precursor occurred in its loop region.

### Unstable secondary structure and weak conservation of *miR529* sequences

**Fig 2:**
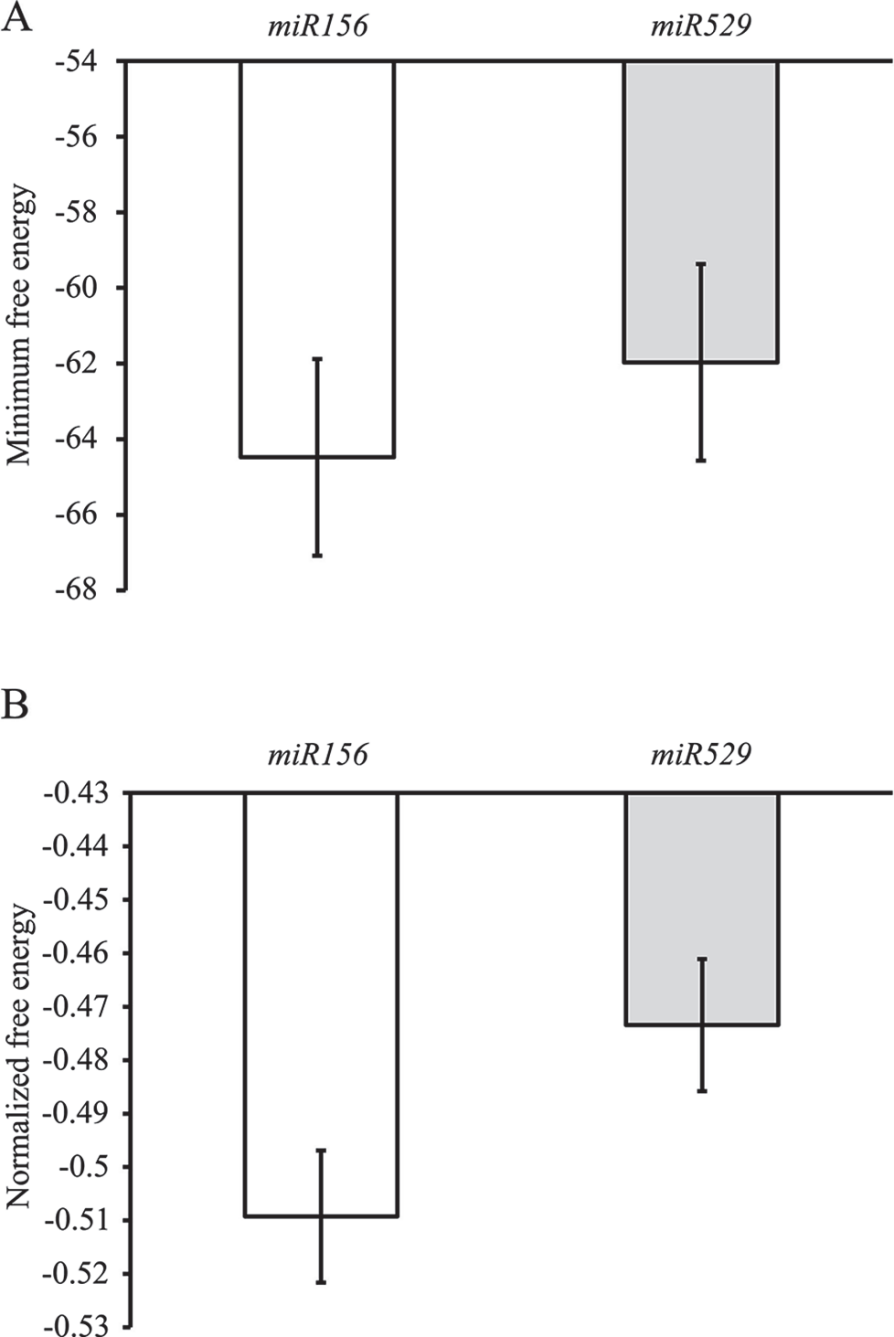
Energetic properties of the secondary structure of *miR156* and *miR529* precursors. Minimum free energy (MFE) (A) and Normalized minimum free energy (NMFE) (B). Error bars indicate the standard error of the mean.

According to the above analyses, we knew that *miR529* shows higher evolutionary rate than *miR156* in its precursor sequence; however the difference is small (Fig 1). Therefore, it requires additional investigation to seek out reliable evidence. Structural studies revealed that the rapid evolution of miRNA precursors greatly affects the stability of secondary structure by introducing sequence variations. With this idea, we separately predicted the minimum free energy (MFE) of secondary structures for pre-*miR529* and pre-*miR156* using RNAfold [19]. Our thermodynamic data show that pre-*miR529* sequences, on average, form more unstable secondary structures than pre-*miR156* sequences, which generally have more negative MFEs (p<0.01) (Fig 2A). After normalizing the MFEs for sequence length, the normalized MFEs (NMFE) showed the same trend as described above (Fig 2B).

In addition, sequence conservation can be taken as a good indicator of evolutionary rate: the slower the sequence evolution, the higher the sequence conservation. The opposite situation is also true. For precursors, we pairwisely compared full-length sequences of *miR156* and *miR529*. As shown in S1A Fig, the precursor sequence identity of *miR156* was higher than that of *miR529* (p<0.01). When only the mature sequences were considered, the difference of sequence identity between *miR156* and *miR529* was considerably dramatic. Pairwise sequence identity was over 95% and 77% for mature *miR156* and *miR529*, respectively (S1B Fig). Therefore, these results provide additional evidence to support that *miR156* family members were more evolutionarily constrained with a slow rate, whereas *miR529* family members evolved more rapidly, particularly in the mature region of the precursor sequence.

### Rapid rate of gene loss in *miR529* after same duplication event in rice

**Fig 3:**
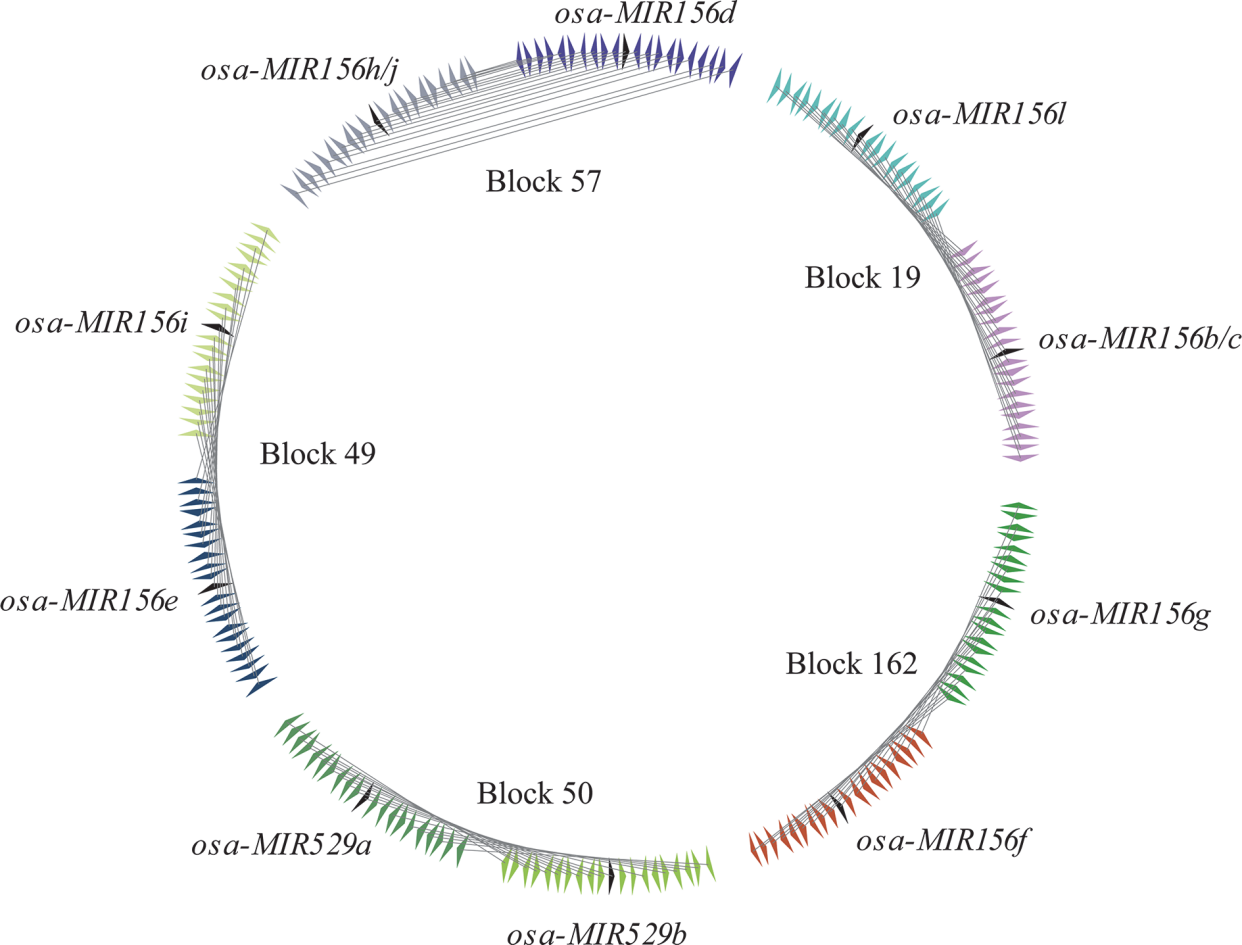
Syntenic duplicated paralogs of *miR156* and *miR529* genes in the duplicated blocks in rice. Black arrowheads indicate the positions of duplicated miRNA pairs in every duplicated block. The flanking protein-coding gene pairs are linked by grey lines.

Previous studies suggest that tandem and segmental duplications dominated the expansion of the *miR156* family [28,29]. As an ancient family, it is reasonable to hypothesize that *miR529* has undergone a history of expansion events similar to *miR156*, which may be the underlying mechanism behind the amplification and diversification of this family. Therefore, we tested this hypothesis that duplication events played a role in the evolution of the *miR529* family. As shown in Fig 3, ten of eleven miRNA genes within *miR156* family formed duplicated pairs and were distributed in four duplicated blocks (blocks 19, 49, 57, and 162). This observation agreed with previous reports suggesting that the *miR156* family mainly arose from large scale segmental duplication [29]. As for the *miR529* family, two *miR529* family members (*miR529a* and *miR529b*) reside within the duplicated block 50, indicating that *miR529* genes also originated from segmental duplication. In addition, two duplicated gene pairs (*miR156b*/*miR156c* and *miR156h* /*miR156j*) of the *miR156* family are located within the same gene and have been characterized as tandem duplications. However, the tandem duplication cannot be inferred because the two *miR529* members in rice were characterized as segmental duplicated pairs. As a result, we investigated whether tandem duplication was the mechanism by which multiple members of the *miR529* family arose in moss, though its genome is not well annotated. Our results indicate that three *miR529* gene pairs of seven genes were located in neighboring regions and the distance between each miRNA pair was less than 100 nt, which satisfies the criteria for tandem duplication (data not shown). Consequently, we can infer that similar duplication mechanisms, including segmental duplication and tandem duplication are at work in the evolution of the *miR529* family, as is the case with the *miR156* family.

In addition, we used synonymous silent substitutions per site (Ks) as a proxy for time to estimate the approximate time of occurrence of the segmental duplication events. As can be seen from Table 1, the mean Ks values of the duplicated genes in block 50 in which *miR529* resides are almost at the same level as the Ks values calculated for the other four blocks. This means that the segmental duplication events involving *miR156* and *miR529* genes occurred approximately 70 million years (Myr) ago, which is consistent with the time at which genome duplication events took place in rice [26,30]. Therefore, these results suggested that *miR156* and *miR529* genes produced in the same genome duplication.

**Table 1:** Identified duplicated blocks containing *miR156* and *miR529* and estimation of the absolute date for segmental duplication events in rice.

Duplicated pair	Block	Mean Ks	SD Ks	Date (Myr)
*osa-mir156bc/l*	19	0.798	0.053	66.159
*osa-mir156e/i*	49	0.764	0.031	63.368
*osa-mir529a/b*	50	0.863	0.026	71.570
*osa-mir156d/hj*	57	0.821	0.032	68.102
*osa-mir156f/g*	162	0.784	0.108	65.030

However, some genes in the duplicated blocks were frequently lost after the genome-wide duplication. For example, *miR156b* and *miR156c* are tandem duplications, yet there is only one corresponding miRNA, *miR156l*, residing within duplicated block 19. To help estimate the loss rate of the *miR156* and *miR529* genes, we tailed the number of flanking protein-coding genes of one miRNA copy that does not have its counterparts in the other duplicated copy within the same block. Then, the rates of gene loss were compared between blocks and between two copies of the same block. As seen in Table 2, the block 50 where *miR529* genes resided had a higher average rate of loss than other four duplicated blocks (blocks 19, 49, 57, and 162) in which *miR156* genes are located (Table 2). In addition, we found that two copies of *miR529* duplicated pairs have greatly asymmetric rates of gene loss. One copy (copy 2) showed the highest percentage (70%) of gene loss, while the other copy had only 28% of gene loss. By contrast, the majority of *miR156* duplicated gene pairs showed very little difference of the gene loss rate between the two copies (Table 2). All together, our results indicate that two *miR529* and miR*156* families experienced the same genome-wide duplication event, and yet exhibited the different rate of gene loss. The rapidly evolving *miR529* family had more asymmetric rate of gene loss between the two copies with a higher average rate of loss than *miR156* family.

**Table 2:** Gene loss rates in duplicated blocks containing *miR156* and *miR529* genes.

Family	Block	Copy 1	Copy 2	Average
miR156	19	0.488	0.416	0.452
miR156	49	0.414	0.479	0.446
miR529	50	0.283	0.701	0.492
miR156	57	0.337	0.585	0.461
miR156	162	0.407	0.505	0.456

## Discussion

How do complex regulatory networks evolve and how does their evolution result in phenotypic change and speciation? For a long time, evolutionary biologists have been devoted to solving these questions. A good approach to understanding the evolution of the gene regulation network is to synthesize the information about all individual regulators and their targets. In our previous study, the SBP-box genes targeted by *miR529* have been found to evolve differently from those targeted by *miR156* in plants [6]. However, the evolutionary patterns of their two combinational regulators, *miR156* and *miR529* are still unknown in plants. Growing evidence has revealed that *miR156* and *miR529* exhibit significant differences in gene expression patterns, taxonomic distribution, and the number of members in the miRNA family (See details in Introduction section). These different characteristics between *miR156* and *miR529* drove us to explore whether they evolve in different patterns in plants.

Our results suggest that narrowly expressed *miR529* genes have higher evolutionary rates in precursor sequences than broadly expressed *miR156* genes. The difference in evolutionary rate between them was small, but additional evidence found by analyzing the structural stability and sequence conservation support this small difference (Fig 1). Therefore, the expression patterns of *miR156* and *miR529* genes were negatively correlated with their respective rates of sequence evolution. This negative correlation between expression patterns and evolutionary rates has also been found in Long Intergenic Noncoding RNAs (LncRNAs) [31], indicative of a general feature of gene evolution. In parallel, the difference of their expression patterns in specific tissues and developmental stages facilitated the combinatorial regulation of their common target genes during evolution.

Our results also revealed that mature miRNA and loop sequences in four miRNA precursor elements change at different rates in the two analyzed miRNA families. The loop sequences of *miR529* have a lower evolutionary rate than those of *miR156* and the opposite trend was observed in the mature miRNA sequences (Fig 1). A large body of evidence has proven that variations in the loop sequences of miRNA precursors can contribute to phenotypic variations by altering miRNA regulation. Wang et al. [32] found that a GG/AA polymorphism in the loop structure of *miR2923a* was correlated to the seed length of two cultivated rice varieties: *japonica* and *indica*. Another study revealed that the alterations in pre-*miR181* loop sequences can modulate the activities of *miR181* family members, albeit of nearly identical or identical mature miRNAs [2]. Moreover, the studies illustrated that the loop region can affect miRNA gene levels by influencing miRNA processing [33]. Accordingly, the question of whether the more variable *miR156* loop sequences may have changed the regulatory activity of its family members by impairing miRNA processing is worthy of additional investigation. On the other hand, variations in mature miRNA can disrupt base pairing to the miRNA* and target sequences. First, the mismatches between miRNA and miRNA* introduced by variations in the mature miRNA sequence can destabilize the structure of the miRNA precursor. This has been confirmed by our analysis of the thermodynamic stability of miRNA precursors (Fig 2A and 2B). Similarly, variations in the mature sequence also disrupt binding to the target sequence. In our previous study, *miR529* targets are more conserved and evolve at a slower rate than *miR156* targets [6]. In this case, frequent variations only in mature *miR529* sequences may lead to the loss of their target regulation but not *miR156* target regulation. Additionally, studies have demonstrated that the variations in mature miRNA sequences can abolish the production of mature miRNAs by disrupting miRNA processing [34,35]. Thus, the subsequent loss of *miR529* regulation may occur through mutations in the mature miRNA during the evolution of core eudicots. Nevertheless, whether or not *miR529* genes block their own biogenesis by altering the secondary structure of pre-miRNAs is still unresolved and requires further studies.

Gene duplication is a major route of origination for miRNAs. Without exception, two ancient *miR156* and *miR529* families were expanded through the same segmental duplication as well as tandem duplication. However, our data also illustrate that *miR156* and *miR529* genes have significantly different rates of gene loss after duplication. Besides a higher average loss rate, *miR529* genes had an obviously asymmetric rate of gene loss in two duplicated copies (Table 2). This asymmetric acceleration of the evolutionary rate can bring about more mutations in one of the paralogs and lead to its more rapid loss [36–38]. Therefore, the high and asymmetric gene loss rate is a predominant mode of inactivation of duplicated *miR529* genes and contributes to reducing the impact of a genome-wide duplication event on functional redundancy within *miR529* family members. On the contrary, the majority of *miR156* family members with low loss rate could be readily retained after duplication, which contributed to the expansion of this family. Moreover, it is proven that these retained *miR156* family members have functionally diverged and exhibit diverse expression profiles in *Arabidopsis* [28]. Accordingly, the gene duplication and subsequent divergence of *miR156* family members could serve as new sources for functional diversity and confer phenotypic differentiation in development.

Altogether, a combination of the rapid evolution of *miR529* sequence (especially the mature sequence), and the asymmetric rate of gene loss makes up the important evolutionary forces that cause the contraction of this miRNA family. An extreme phenomenon is that all the *miR529* members are extinct in core eudicots. Our previous work demonstrated stronger purifying selection against mutations within the binding sites of *miR529* targets [6]. Meanwhile, these *miR529* targets also are cooperatively controlled by *miR156*. If the mutations occurred on the binding site of *miR529* targets, they would be bound to disrupt ligand binding between *miR156* and this same target. Therefore, the economical and balanced way to change *miR529* combinatorial regulation is to change *miR529* itself. Indeed, the retention of *miR529* binding sites in some members of SBP-box genes but no *miR529* candidate in core eudicots gave it a full interpretation (S2 Table). On the contrary, the increased mutations in *miR156* targets but the relative conservation in *miR156* genes should contribute to harmonizing the *miR156* regulation system. For *miR156*, the variable loop sequence followed by the divergence of duplicated gene pairs after duplication might be the principle manner in which *miR156* functions are enriched. Moreover, accumulating functional studies have revealed that the SBP-box genes targeted by *miR156* and *miR529* are involved in various development processes and lead to many morphological differences, such as leaf, flower, pollen, and fruit (see review [39]). It is well known that these important characteristics might distinguish monocot and dicot from each other. Therefore, the differences between *miR156* and *miR529* regulation mechanisms might contribute to the morphological divergence of monocot and dicot. In conclusion, our data indicate that *miR156* and *miR529* genes show different evolutionary dynamics. *MiR156* genes are continually differentiated and amplified by accelerated mutations in their hairpin loops and diverging duplicated gene pairs after duplication. By contrast, *miR529* genes gradually reduce their abundance via accumulating more mutations in the mature miRNA sequences and by rapidly losing one of the paralogous duplicated pairs soon after the duplication event. Taken together, these results enhance our understanding of the different evolutionary mechanisms driving changes in two miRNA families and the different regulatory mechanisms in the gene regulation network.

## Supporting Information

S1 Fig Comparisons of sequence identity between miR156 and *miR529* genes in precursor (A) and mature (B) sequences. Error bars indicate the standard error of the mean.
(TIF)

S1 Table High-confidence entries of *miR156* and miR529 family used in this study. (DOC)

S2 Table Predicted targets for *miR529* in three eudicots species. (DOC)
