# Supplementary figures and images for: Evolutionary Comparison of Two Combinatorial Regulators of SBP-Box Genes, MiR156 and MiR529, in Plants
Source: PLoS One. 2015 Apr 24;10(4):e0124621. doi: 10.1371/journal.pone.0124621 (PMC4409300; doi:10.1371/journal.pone.0124621)

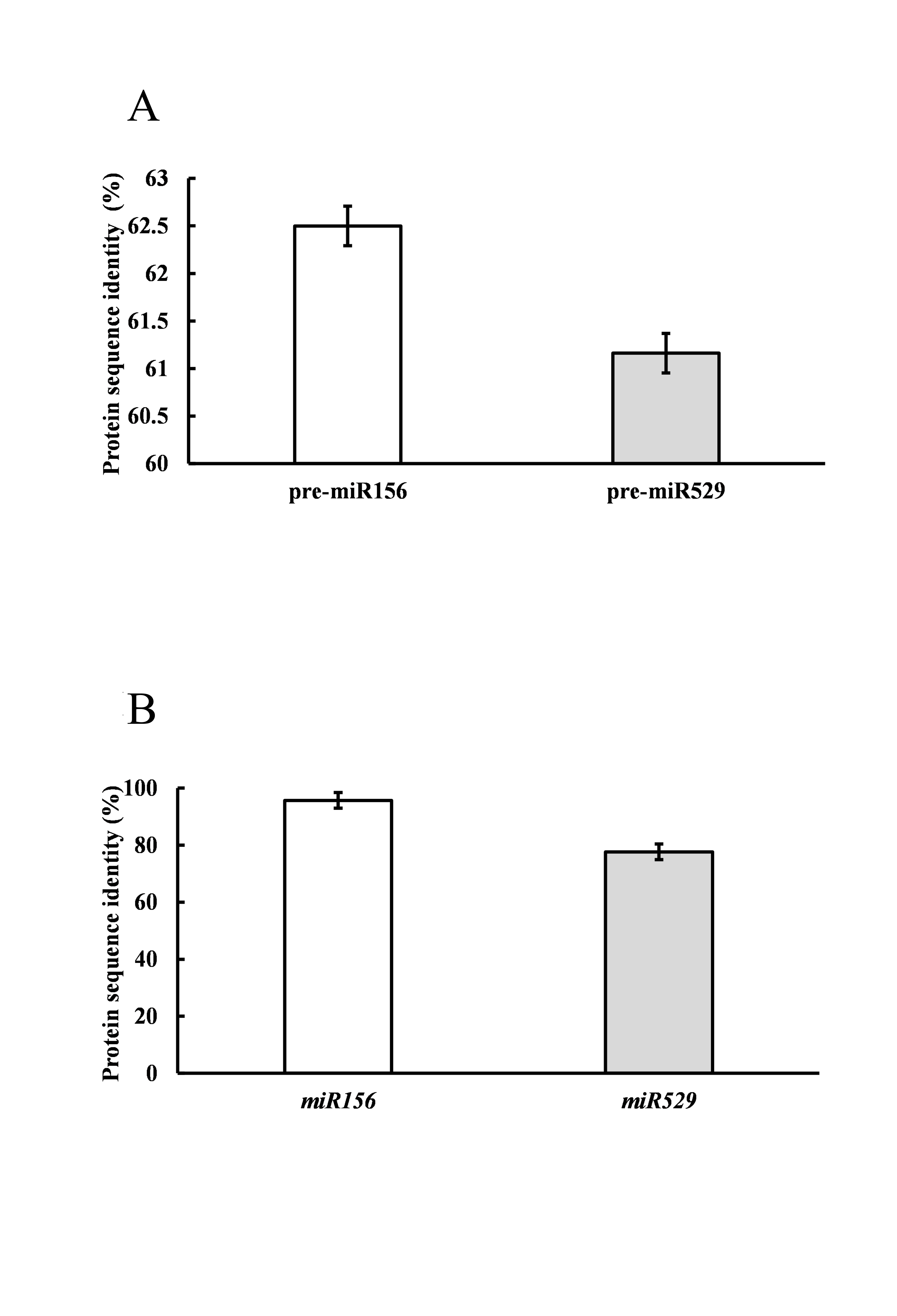

Supplement: S1 Fig — Error bars indicate the standard error of the mean. (TIF) [file pone.0124621.s001.tif]
