# Supplementary material for: Evolutionary Comparison of Two Combinatorial Regulators of SBP-Box Genes, MiR156 and MiR529, in Plants
Source: PLoS One. 2015 Apr 24;10(4):e0124621. doi: 10.1371/journal.pone.0124621 (PMC4409300; doi:10.1371/journal.pone.0124621)
Supplement: S1 Table — (DOC) [file pone.0124621.s002.doc]

**S1 Table. The high-confidence entries of *miR156* and *miR529* family used in this study**

| **Family** | **ID** | **Accession** | **Organism** |
| --- | --- | --- | --- |
| miR156 | *aly-miR156a* | MI0014502 | *Arabidopsis lyrata* |
| miR156 | *aly-MIR156b* | MI0014503 | *Arabidopsis lyrata* |
| miR156 | *aly-MIR156c* | MI0014504 | *Arabidopsis lyrata* |
| miR156 | *aly-MIR156d* | MI0014505 | *Arabidopsis lyrata* |
| miR156 | *ath-MIR156a* | MI0000178 | *Arabidopsis thaliana* |
| miR156 | *ath-MIR156b* | MI0000179 | *Arabidopsis thaliana* |
| miR156 | *ath-MIR156c* | MI0000180 | *Arabidopsis thaliana* |
| miR156 | *ath-MIR156d* | MI0000181 | *Arabidopsis thaliana* |
| miR156 | *ath-MIR156e* | MI0000182 | *Arabidopsis thaliana* |
| miR156 | *ath-MIR156f* | MI0000183 | *Arabidopsis thaliana* |
| miR156 | *ath-MIR156h* | MI0001083 | *Arabidopsis thaliana* |
| miR156 | *hvu-MIR156b* | MI0030546 | *Hordeum vulgare* |
| miR156 | *osa-MIR156a* | MI0000653 | *Oryza sativa* |
| miR156 | *osa-MIR156b* | MI0000654 | *Oryza sativa* |
| miR156 | *osa-MIR156c* | MI0000655 | *Oryza sativa* |
| miR156 | *osa-MIR156d* | MI0000656 | *Oryza sativa* |
| miR156 | *osa-MIR156e* | MI0000657 | *Oryza sativa* |
| miR156 | *osa-MIR156f* | MI0000658 | *Oryza sativa* |
| miR156 | *osa-MIR156g* | MI0000659 | *Oryza sativa* |
| miR156 | *osa-MIR156h* | MI0000660 | *Oryza sativa* |
| miR156 | *osa-MIR156i* | MI0000661 | *Oryza sativa* |
| miR156 | *osa-MIR156j* | MI0000662 | *Oryza sativa* |
| miR156 | *osa-MIR156l* | MI0001091 | *Oryza sativa* |
| miR156 | *sbi-MIR156e* | MI0001856 | *Sorghum bicolor* |
| miR156 | *sbi-MIR156f* | MI0010860 | *Sorghum bicolor* |
| miR156 | *tae-MIR156* | MI0016450 | *Triticum aestivum* |
| miR156 | *zma-MIR156d* | MI0001456 | *Zea mays* |
| miR156 | *zma-MIR156e* | MI0001461 | *Zea mays* |
| miR156 | *zma-MIR156k* | MI0001847 | *Zea mays* |
| miR156 | *ppt-MIR156a* | MI0003506 | *Physcomitrella patens* |
| miR156 | *ppt-MIR156c* | MI0005654 | *Physcomitrella patens* |
| miR156 | *csi-MIR156* | MI0016691 | *Citrus sinensis* |
| miR156 | *nta-MIR156c* | MI0021321 | *Nicotiana tabacum* |
| miR156 | *nta-MIR156g* | MI0021325 | *Nicotiana tabacum* |
| miR156 | *nta-MIR156h* | MI0021326 | *Nicotiana tabacum* |
| miR156 | *nta-MIR156i* | MI0021327 | *Nicotiana tabacum* |
| miR156 | *nta-MIR156j* | MI0021328 | *Nicotiana tabacum* |
| miR156 | *ptc-MIR156e* | MI0002188 | *Populus trichocarpa* |
| miR156 | *ptc-MIR156f* | MI0002189 | *Populus trichocarpa* |
| miR156 | *ptc-MIR156j* | MI0002193 | *Populus trichocarpa* |
| miR156 | *vvi-MIR156b* | MI0006486 | *Vitis vinifera* |
| miR156 | *vvi-MIR156d* | MI0006488 | *Vitis vinifera* |
| miR156 | *vvi-MIR156g* | MI0006491 | *Vitis vinifera* |
| miR529 | *bdi-MIR529* | MI0018229 | *Brachypodium distachyon* |
| miR529 | *osa-MIR529a* | MI0003202 | *Oryza sativa* |
| miR529 | *osa-MIR529b* | MI0005804 | *Oryza sativa* |
| miR529 | *zma-MIR529* | MI0013241 | *Zea mays* |
| miR529 | *ppt-MIR529a* | MI0005921 | *Physcomitrella patens* |
| miR529 | *ppt-MIR529b* | MI0005922 | *Physcomitrella patens* |
| miR529 | *ppt-MIR529c* | MI0005923 | *Physcomitrella patens* |
| miR529 | *ppt-MIR529d* | MI0005924 | *Physcomitrella patens* |
| miR529 | *ppt-MIR529e* | MI0005925 | *Physcomitrella patens* |
| miR529 | *ppt-MIR529f* | MI0005926 | *Physcomitrella patens* |
| miR529 | *ppt-MIR529g* | MI0005927 | *Physcomitrella patens* |

Note：All data used in this study can be downloaded from the public miRBase database (release 21, [http://www.mirbase.org/](http://www.mirbase.org/).)) operated by the University of Manchester.
