# Supplementary material for: Evolutionary Comparison of Two Combinatorial Regulators of SBP-Box Genes, MiR156 and MiR529, in Plants
Source: PLoS One. 2015 Apr 24;10(4):e0124621. doi: 10.1371/journal.pone.0124621 (PMC4409300; doi:10.1371/journal.pone.0124621)
Supplement: S2 Table — (DOC) [file pone.0124621.s003.doc]

**S2 Table.** The predicted targets for *miR529* in three eudicots species

| **Organism** | **Target gene** | **Target Sequence** |
| --- | --- | --- |
| *Arabidopsis* | AT1G27360 | CACCGUGCUCUCUCUCUUCU |
| *Arabidopsis* | AT1G27370 | UACAGUGCUCUCUCUCUUCU |
| *Arabidopsis* | AT2G42200 | AACUGUGCUCUCUCUCUUCU |
| *Arabidopsis* | AT3G57920 | AGCUGUGCUCUCUCUCUUCU |
| *Arabidopsis* | AT5G43270 | GAUGGUGCUCUCUCUCUUCU |
| *Arabidopsis* | AT5G50570 | GAUUGUGCUCUCUCUCUUCU |
| *Arabidopsis* | AT5G50670 | GAUUGUGCUCUCUCUCUUCU |
| Grape | GSVIVG01008556001 | AGAUUGUGCUCUCUCUCUUCU |
| Grape | GSVIVG01008556001 | GAUUGUGCUCUCUCUCUUCU |
| Grape | GSVIVG01010496001 | CGUCGUGCUCUCUCUCUUCU |
| Grape | GSVIVG01010522001 | GAUCGUGCUCUCUCUCUUCU |
| Grape | GSVIVG01012247001 | AAUUGUGCUCUCUCUCUUCU |
| Grape | GSVIVG01018205001 | AGGACGUGCUCUCUCUCUUCU |
| Grape | GSVIVG01018205001 | GGACGUGCUCUCUCUCUUCU |
| Grape | GSVIVG01018205001 | GGAC-GUGCUCUCUCUCUUCU |
| Grape | GSVIVG01033064001 | GGACUGUGCUUUCUCUCUUCU |
| Grape | GSVIVG01033519001 | AAGCUGUGCUCUCUCUCUUCU |
| Grape | GSVIVG01033519001 | AGCUGUGCUCUCUCUCUUCU |
| *Poplus* | Potri.001G058600 | AACCGUGCUCUCUCUCUUCU |
| *Poplus* | Potri.002G142400 | AGGAUGUGCUCUCUCUCUUCU |
| *Poplus* | Potri.002G142400 | GGAUGUGCUCUCUCUCUUCU |
| *Poplus* | Potri.003G169400 | AACCGUGCUCUCUCUCUUCU |
| *Poplus* | Potri.008G097900 | AGCUGUGCUCUCUCUCUUCU |
| *Poplus* | Potri.010G154300 | AGCUGUGCUCUCUCUCUUCU |
| *Poplus* | Potri.014G057800 | AGGAUGUGCUCUCUCUCUUCU |
| *Poplus* | Potri.014G057800 | GGAUGUGCUCUCUCUCUUCU |
| *Poplus* | Potri.015G098900 | GGAUUGUGCUCUCUCUCUUCU |
| *Poplus* | Potri.016G048500 | AGCUGUGCUCUCUCUCUUCU |
| *Poplus* | Potri.016G048500 | GAGCUGUGCUCUCUCUCUUCU |

Note: Due to no *miR529* paralogs in eudicots, we adopted the mature *miR529* sequences from other species used in this study to predict their binding sites on SBP-box gene transcripts of three eudicots species: Arabidopsis, grape and poplar. High-confidence prediction of *miR529* targets was performed by psRNATarget server (http: //bioinfo3.noble.org.psRNATarget/) with default settings and the more strict empirical parameters were used to filter them as previously described by Ling .

References:

Dai, X. and Zhao P. X. 2011. psRNATarget: a plant small RNA target analysis server. *Nucleic Acids Research* 39(Web Server issue): W155-159.

Ling, L. Z. and Zhang S. D. (2012). Exploring the evolutionary differences of SBP-box genes targeted by *miR156* and *miR529* in plants. *Genetica* 140(7-9): 317-324.
